# *AlphaFold*-assisted structure determination of a bacterial protein of unknown function using X-ray and electron crystallography

**DOI:** 10.1107/S205979832400072X

**Published:** 2024-03-07

**Authors:** Justin E. Miller, Matthew P. Agdanowski, Joshua L. Dolinsky, Michael R. Sawaya, Duilio Cascio, Jose A. Rodriguez, Todd O. Yeates

**Affiliations:** a Molecular Biology Institute, University of California, Los Angeles, Los Angeles, CA 90095, USA; b Department of Chemistry and Biochemistry, University of California, Los Angeles, Los Angeles, CA 90095, USA; c Institute for Genomics and Proteomics, UCLA–DOE, Los Angeles, CA 90095, USA; University of Queensland, Australia

**Keywords:** electron diffraction, protein structure prediction, *AlphaFold*, bacterial proteins, molecular replacement

## Abstract

Macromolecular crystallography generally requires the recovery of missing phase information from diffraction data to reconstruct an electron-density map of the crystallized molecule. Most recent structures have been solved using molecular replacement as a phasing method, requiring an *a priori* structure that is closely related to the target protein to serve as a search model; when no such search model exists, molecular replacement is not possible. New advances in computational machine-learning methods, however, have resulted in major advances in protein structure predictions from sequence information. Methods that generate predicted structural models of sufficient accuracy provide a powerful approach to molecular replacement. Taking advantage of these advances, *AlphaFold* predictions were applied to enable structure determination of a bacterial protein of unknown function (UniProtKB Q63NT7, NCBI locus BPSS0212) based on diffraction data that had evaded phasing attempts using MIR and anomalous scattering methods. Using both X-ray and micro-electron (microED) diffraction data, it was possible to solve the structure of the main fragment of the protein using a predicted model of that domain as a starting point. The use of predicted structural models importantly expands the promise of electron diffraction, where structure determination relies critically on molecular replacement.

## Abstract 2

The structure determination of a small protein of unknown function by molecular replacement using a search model predicted by new machine-learning methods is reported. Notably, the approach was successful using electron diffraction data collected from a protein microcrystal, highlighting a potentially important new route for structure determination.

## 1. Introduction

New variations on traditional X-ray crystallography are expanding the power of diffraction methods for macromolecular structure determination (Thompson *et al.*, 2020 ▸; Johansson *et al.*, 2017 ▸; Martynowycz & Gonen, 2018 ▸; Xu *et al.*, 2019 ▸; Clabbers *et al.*, 2021 ▸; Terwilliger *et al.*, 2023 ▸). Two ongoing developments are notable for their potential scope. Firstly, recent algorithmic advances in protein structure prediction have made it possible, in many cases, to generate three-dimensional models that are accurate enough for molecular-replacement protocols (Terwilliger *et al.*, 2023 ▸; Jumper *et al.*, 2021 ▸; Baek *et al.*, 2021 ▸; Giorgetti *et al.*, 2005 ▸). Such cases ultimately allow an experimental structure to be elucidated without the need for experimental phasing (*i.e.* heavy-atom or anomalous approaches) and without prior experimental knowledge of a similar protein structure. Secondly, on the side of experimental advances, electron-based diffraction is attracting attention as a potential approach that is suitable for very small crystals (Nannenga & Gonen, 2018 ▸). These two lines of exploration intersect. Anomalous scattering methods of phasing do not readily transfer to electron diffraction, and traditional isomorphous replacement methods are likewise challenged by lower electron scattering factors for heavy atoms. These challenges elevate the importance of molecular replacement for solving structures using electron diffraction data, including with predicted models. More case studies are needed to demonstrate the utility, and the challenges, of these new structure-determination approaches.

**Figure 1:**
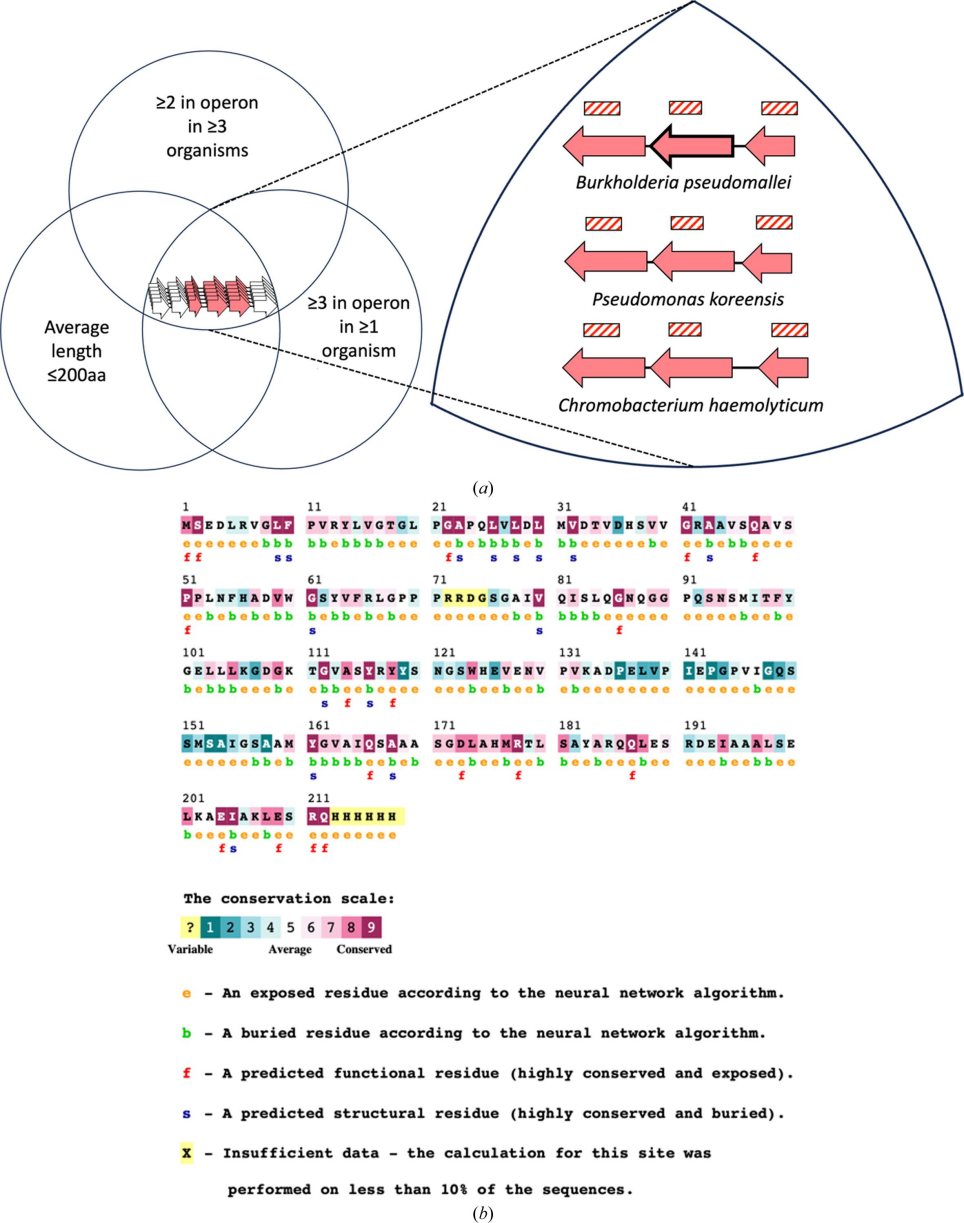
Representation of the criteria used to select genes encoding proteins with an elevated likelihood of self-assembly (including Q63NT7). (*a*) Graphical representation of the rationale for structural investigations on Q63NT7, where the selection criteria are depicted as a Venn diagram as in Beeby *et al.* (2009 ▸). Several representative operons with respective organisms of origin obeying the selection criteria are highlighted on the right. The Q63NT7-encoding gene is depicted by a bold arrow. DUF1843-containing genes are shown as red arrows and nonhomologous genes are shown as white arrows. (*b*) Graphical representation of the per-residue conservation of Q63NT7 using *ConSurf* (Ashkenazy *et al.*, 2016 ▸).

The subject of the present study is a bacterial protein of unknown structure and function: UniProtKB Q63NT7. It was chosen for structural investigation based on its unusual genomic presentation. The tendency of the PF08898 protein family (proteins containing the domain DUF1843) to be encoded as repeated paralogs within individual operons suggested that it might form part of a larger self-assembling protein complex, as proposed in an earlier bioinformatics study (Beeby *et al.*, 2009 ▸; Fig. 1 ▸ *a*), but no structural data were available. Biochemical and structural studies were therefore undertaken to investigate the structure of this protein domain and to evaluate whether it might form a larger self-assembling complex. Difficulties in obtaining large crystals led to expanded efforts, including structure determination from small crystals by electron diffraction and molecular replacement using predicted models.

## 2. Materials and methods

### 2.1. Gene synthesis

Codon-optimized gene sequences were ordered from Integrated DNA Technologies or Twist Biosciences with overlapping sequences corresponding to flanking regions around the HindIII and NdeI restriction sites in the pET-22b expression vector. Intergenic sequences for two-component designs were taken from a pETDuet-1 expression plasmid and ordered as a single gene fragment. Macromolecule-production information is summarized in Supplementary Table S1.

### 2.2. Protein expression and purification

Designs were cloned into pET-22b expression vectors using Gibson assembly. The correct cloning of the gene was verified by Sanger sequencing. Small-scale expression was performed in *Escherichia coli* BL21(DE3) cells grown in 200 ml cultures using auto-induction medium grown for 24 h at 25°C. Cells were lysed in 50 m*M* Tris–HCl pH 8.0, 250 m*M* NaCl supplemented with 5 m*M* β-mercaptoethanol and EDTA-free protease-inhibitor tablets (Thermo Fisher Scientific) using an Emulsiflex C3 homogenizer and affinity purified using Ni–NTA agarose resin (Thermo Fisher Scientific) in a gravity-flow column. Protein was washed with lysis buffer plus 100 m*M* imidazole and eluted in lysis buffer plus 500 m*M* imidazole. Eluted protein was dialyzed against imidazole overnight at 4°C. Samples were run on SDS–PAGE to assess purity before size-exclusion chromatography (SEC) using a Superdex 75 column (Cytiva Life Sciences) attached to an ÄKTA FPLC (Cytiva Life Sciences). Sodium azide was then added to SEC elution fractions at a concentration of 0.05% as well as EDTA at a concentration of 5 m*M*.

### 2.3. Crystallization

96-well crystal screens were set up using a Mosquito liquid handler (SPT Labtech) in hanging-drop vapor-diffusion format. The trays were incubated at 22°C until crystals were observed. Cubic crystals of form I appeared after ∼6 months in conditions consisting of 100 m*M* bis-Tris pH 5.5, 25% PEG 3350 with 20 mg ml^−1^ protein. Form 2 crystals appeared within a week in conditions consisting of 100 m*M* bis-Tris pH 5.5, 100 m*M* ammonium acetate, 17% PEG 10 000 with 20 mg ml^−1^ protein. Form 3 crystals grew at a protein concentration of 100 mg ml^−1^ in 100 m*M* Tris–HCl pH 8.5, 150 m*M* MgCl_2_, 12.5% PEG 8000. Both form 2 and form 3 crystals were directly frozen in liquid nitrogen without any additional cryoprotectant for data collection at 100 K. Crystallization information is summarized in Supplementary Tables S2, S3 and S4.

### 2.4. X-ray data collection and processing

X-ray diffraction data sets were collected on NE-CAT beamline 24-ID-C at the Advanced Photon Source (APS) equipped with an EIGER 16M detector and on beamline 24-ID-E equipped with a PILATUS 6M-F detector. The *XDS* package was used to index the diffraction data (Kabsch, 2010 ▸). Diffraction data statistics are provided in Table 1 ▸.

### 2.5. Negatively stained transmission electron microscopy (EM)

Crystal drops containing crystals were diluted in 5 µl distilled water and mixed using a pipette. 3 µl was applied onto glow-discharged Formvar/Carbon 300 mesh copper grids (Ted Pella) for 60 s. Excess sample was wicked using filter paper and the grid was immediately washed twice with distilled water. A 2% uranyl acetate solution was applied to the grid and then immediately wicked using filter paper. A final incubation of the grid with 2% uranyl acetate was performed for 20 s and the grid was dried completely using filter paper. Imaging was performed on Tecnai T12 and Talos F200C microscopes (Thermo Fisher).

### 2.6. Micro-ED data collection

Crystal drops containing crystals were diluted in 5 µl mother liquor, without the addition of cryoprotectant, from the crystal reservoir and mixed gently using a pipette. 5 µl was applied onto glow-discharged Quantifoil 300 mesh 2/2 copper grids (Electron Microscopy Sciences) and vitrified using a Vitrobot Mark IV with pre-wetted blotting paper (Thermo Fisher). Seven movies were collected from unique crystals on a Tecnai TF30 microscope (Thermo Fisher) fitted with a TVIPS TemCam-F416 and a single-tilt cryo-transfer holder (Gatan) employing a maximum tilt range of −60° to +60°. Continuous-rotation microED data were collected at a rotation rate of 0.085° s^−1^. Diffraction data were indexed using the *XDS* package (Kabsch, 2010 ▸) and scaled using *XSCALE* (Kabsch, 2010 ▸). Diffraction data statistics are provided in Table 1 ▸.

### 2.7. Molecular replacement and structure refinement

*Phaser* (McCoy *et al.*, 2007 ▸) was used for molecular replacement. *AlphaFold* (Jumper *et al.*, 2021 ▸) was used to generate molecular-replacement search models. After refining the initial *AlphaFold* search model on the basis of the form 1 diffraction data, we used the refined structure to phase the form 2 and form 3 crystals, as this gave the best statistics and resulted in the best electron-density maps. *Coot* (Emsley *et al.*, 2010 ▸) was used for model building, and refinement was performed using *Phenix* (Liebschner *et al.*, 2019 ▸). Atomic refinement statistics are provided in Table 2 ▸.

## 3. Results

### 3.1. Protein expression and purification

**Figure 2:**
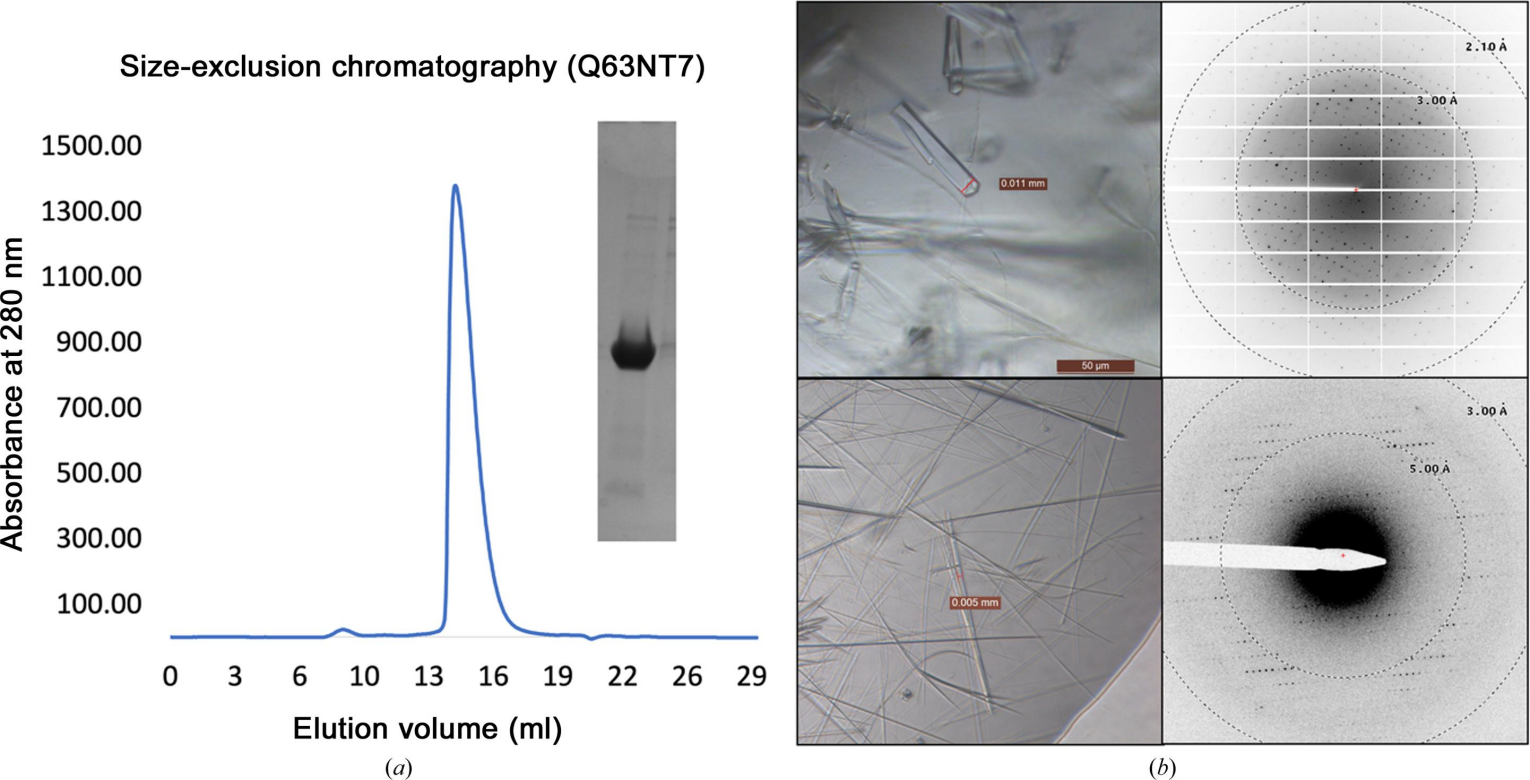
Biochemical characterization of the Q63NT7 protein: (*a*) SEC and SDS–PAGE reveal the homogeneity and high purity of the Q63NT7 protein. (*b*) Form 1 (top) and form 2 (bottom) crystals and representative diffraction data collected using an X-ray source or electron microscope, respectively (see Section 2).

The Q63NT7 protein from *Burkholderia pseudomallei* is 212 amino acids in length (molecular mass 22.5 kDa). It contains two predicted domains: an N-terminal domain of unknown function (14.5 kDa; DUF1842) and the aforementioned C-terminal domain (DUF1843; 5.4 kDa), which tends to appear in multiple paralogous copies within individual bacterial operons. We ordered sequences encoding the Q63NT7 sequence with C-terminal 6×His tags. We expressed the protein recombinantly in *E. coli* BL21(DE3) cells (Supplementary Table S1). Biochemical characterization of this protein suggested that the protein is monodisperse and is likely to be monomeric in solution (Fig. 2 ▸ *a*).

### 3.2. Protein crystallization and crystal forms

Encouraged by the purity of our protein sample, we attempted to solve the structure crystallographically. Q63NT7 presented a challenge for obtaining large, well ordered crystals. This led us to explore multiple distinct crystal forms with the goal of improving the diffraction quality and, as discussed later, to attempt to visualize a substantial region of the protein that could not be resolved in density maps.

Initial crystallization trials yielded abundant needles across many crystallization conditions, but attempts to obtain X-ray diffraction data were unsuccessful. We also observed inconsistent crystal formation across our replicated crystal trays. Even so, we were ultimately (after approximately six months) able to optimize these conditions and grow larger rectangular-shaped crystals that diffracted beyond 3 Å resolution on a synchrotron microfocus beamline (Fig. 2 ▸ *b*). We collected data sets from these crystals, which we refer to as form 1. Diffraction data indexing revealed the space group to be *P*2_1_ (Table 1 ▸). The highest resolution resulted from data collected from a single crystal specimen.

**Figure 3:**
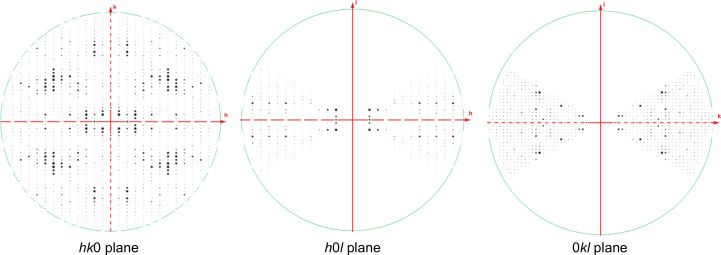
Slices through reciprocal space show the missing cone that is present in microED data collected from form 2 crystals. Principal zones are shown to illustrate the missing cone of data due to preferred orientation of crystals on the grid.

In parallel with efforts to phase data from form 1 crystals, we sought to achieve higher quality diffraction from Q63NT7 crystals. Anticipating that needle-shaped crystals might be especially suitable for micro-electron diffraction (microED) methods owing to their limited thickness, after failing to obtain X-ray diffraction data using microfocus or traditional beamlines, we used an electron microscope to investigate the order and diffraction quality of the needle-shaped microcrystals that grew in showers in some of our drops. We first pipetted those drops onto Formvar/Carbon electron microscopy grids, stained them with uranyl acetate and imaged them. The crystallinity of our sample was evident by the appearance of lattice lines in the sample (Supplementary Fig. S1). We proceeded to vitrify microcrystals under similar conditions to investigate their suitability for diffraction experiments. These microcrystals typically diffracted to 3 Å resolution in an electron microscope operating in diffraction mode (see Section 2; Fig. 2 ▸ *b*, bottom panel). We collected diffraction data sets from four microcrystals. The crystal unit-cell dimensions were non-isomorphous with those of form 1 crystals (Table 1 ▸), so we refer to these as form 2 crystals. Unfortunately, the crystals, which appeared to be ribbon-shaped at high magnification, suffered from preferred orientation problems and did not diffract at high tilt angles beyond ±30°, possibly due to increased thickness in the diffracting dimension when tilted. This led to us only acquiring 59% completeness in a merged diffraction data set. Furthermore, it was difficult to confidently assign a space group due to a substantial missing cone of reflections (Fig. 3 ▸). The quality of the individual data sets was poor, partly owing to weaker signal (for example unsatisfactory *R*_sym_ values) from some specimens; merging multiple data sets did not substantially improve the data quality, but improved the completeness slightly. We therefore elected to proceed using a data set obtained by merging diffraction from four crystals. Unfortunately, since the regions of reciprocal space missing from the distinct data sets were largely overlapping, the final data set was only 59% complete. Owing to the lack of data along the c* axis, systematic absences were difficult to discern from missing data, and the number of 2_1_ screw axes was initially unclear. Especially along the b* axis, the intensity magnitudes of the odd-index reflections were higher than expected based on the noise level (Supplementary Fig. S2). This suggests the possibility of measurable dynamical scattering effects in electron diffraction experiments, as has been discussed (Nannenga *et al.*, 2018 ▸; Nannenga & Gonen, 2014 ▸). Attempts at molecular replacement with the form 2 electron diffraction data in space groups 16, 17, 18 and 19 ultimately confirmed that *P*2_1_2_1_2_1_ was correct for form 2 microcrystals based on a much higher LLG value.

Finally, we continued to optimize the crystallization conditions and identified another condition that grew well diffracting needle-shaped crystals that were suitable for data collection on the synchrotron microfocus beamline (Supplementary Fig. S3). We were able to collect a complete data set from a single crystal, which could be processed in space group *P*2_1_2_1_2_1_. We refer to this crystal as form 3, since its unit-cell dimensions were distinct from those of forms 1 and 2 (Table 1 ▸).

### 3.3. Molecular replacement using *AlphaFold* models

Efforts to phase the highest quality data set (form 1) using experimental techniques did not lead to immediate success; selenomethionine-labeled protein crystals did not diffract, and we observed no heavy-atom signal in the diffraction patterns of crystals soaked in caesium chloride or potassium iodide.

**Figure 4:**
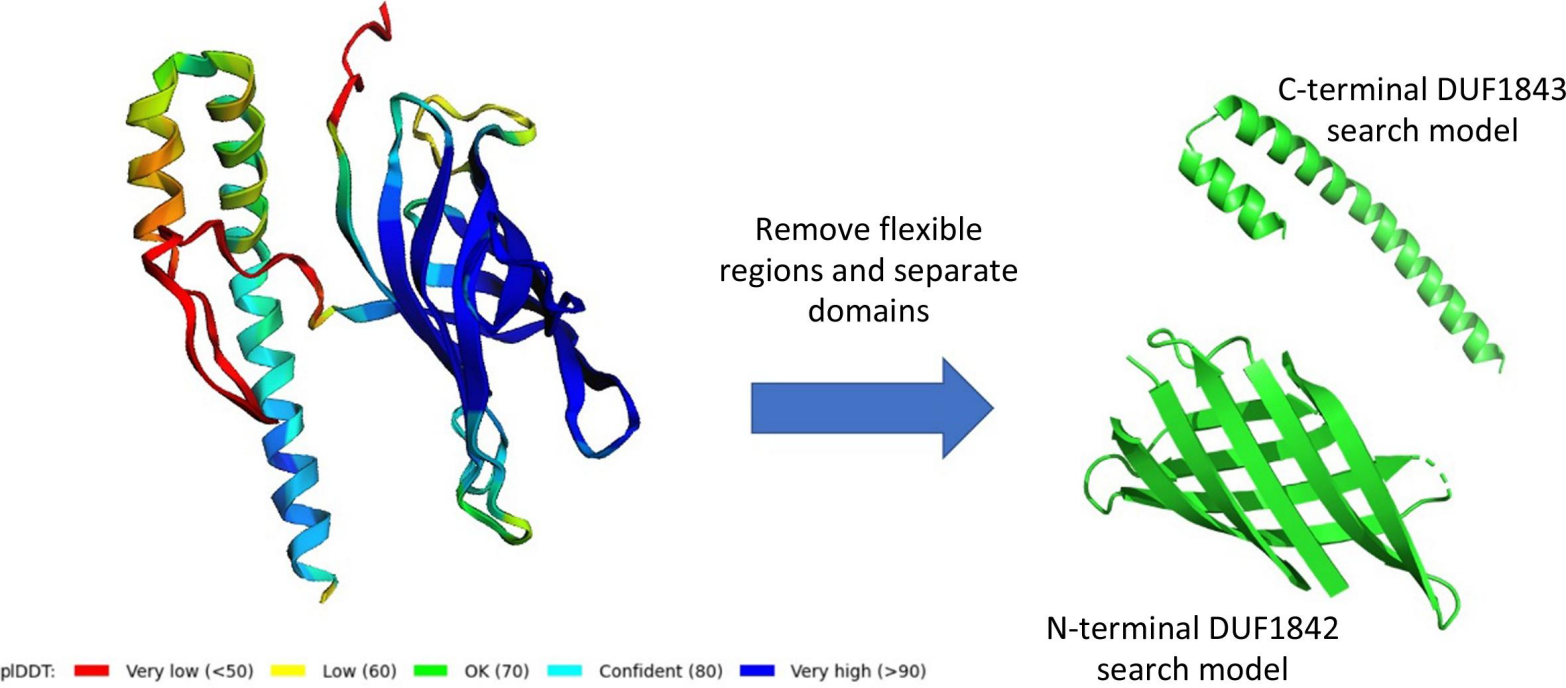
*AlphaFold* model of the Q63NT7 protein used as a molecular-replacement search model. pLDDT gives a per-residue metric of confidence in model prediction.

Inspired by studies that had used *AlphaFold* models to phase data sets with little *a priori* information, we used the software to generate a model of Q63NT7 (Fig. 4 ▸). *AlphaFold* identified two domains in the protein joined by a long linker. The N-terminal domain was predicted to fold into a β-barrel composed of eight antiparallel strands. *AlphaFold* predicted this domain with a high degree of confidence based on per-residue pLDDT scores. The C-terminal domain was predicted to form a small helical bundle with modest pLDDT confidence metrics. Applying existing molecular-replacement methods to our *AlphaFold*-based molecular-replacement efforts, we separated the coordinates of the two domains into independent files and removed extended loop segments, including the long linker between the domains (Fig. 4 ▸).

**Figure 5:**
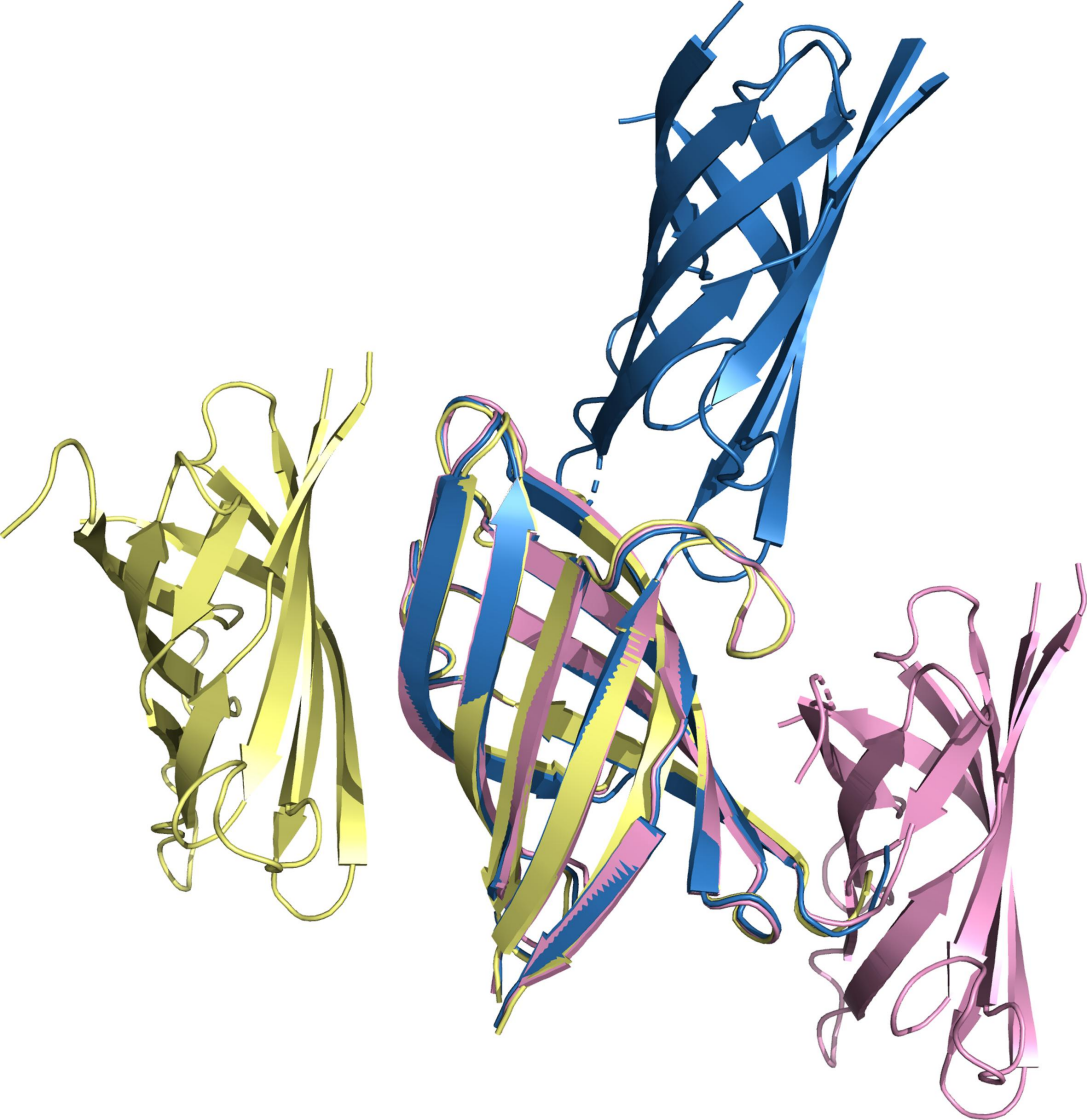
Structural comparison of asymmetric units from three crystal forms. Cartoon representations of the structures solved from form 1 crystals (pink), form 2 crystals (yellow) and form 3 crystals (blue).

We used these two structures, including side chains, as search models for molecular replacement with *Phaser* (McCoy *et al.*, 2007 ▸). Remarkably, data sets from all three crystal forms gave solutions that passed the *Phaser* metrics for a correct solution using the N-terminal β-sheet-rich domain. The solution was further validated using a test search model that excluded six residues in β-strand 5; maps phased from such a molecular-replacement model produced positive density at the expected positions in an *F*_o_ − *F*c difference map (Supplementary Fig. S4). All three crystal forms identified two copies of the N-terminal β-barrel domain in the asymmetric unit (Fig. 5 ▸). Form 1 crystals gave a combined LLG value of 719, form 2 crystals gave an LLG value of 394 and form 3 crystals gave an LLG value of 305. In contrast, none of the crystals could be phased using the C-terminal α-helical domain as a search model using similar program parameters. Given the small contribution of scattering attributed to this domain because of its small size, these negative results were not altogether surprising. Flexibility concerns, which arose later, were also important.

We next investigated whether similar structures existed in the PDB and whether, in retrospect, they too could have served as search models for molecular replacement with our data. To do this, we submitted the structure obtained from the form 1 crystal data set (after molecular replacement and preliminary refinement) to the *DALI* server and identified the top five closest-matching protein folds (*i.e.* those with the highest *Z*-scores) in the PDB (Supplementary Fig. S5; Holm & Laakso, 2016 ▸). Interestingly, the structure that was identified as the most similar to our own based on *Z*-score is an outer membrane protein from *Pseudomonas aeruginosa*. The ensuing molecular-replacement attempts did not produce plausible packing solutions using form 1 data. We went on to test whether these known models would produce solutions for the form 2 data with lower completeness, which might lend themselves to incorrect solutions more than the comparatively better form 1 data. For each of these trials, *Phaser* was unable to produce molecular-replacement statistics indicative of a correct solution. The LLGs for these trials were all below what would be expected for a correct solution, and all were significantly lower than for the *AlphaFold* model: 129 (PDB entry 2erv), 57 (PDB entry 2f1v), 25 (PDB entry 4u8u), 112 (PDB entry 4rcl) and 119 (PDB entry 4bbo). We further tested whether the *FoldSeek* (van Kempen *et al.*, 2023 ▸) search algorithm could be used to identify other molecular-replacement search models, either from the PDB or amongst the vast number of predicted protein models. Performing molecular replacement using our form 2 data, the most similar structure identified using the *FoldSeek* search was PDB entry 6cd8 (the GID4 subunit of a ubiquitin ligase complex), which gave a *Phaser* LLG value of only 47. Our finding was that only the *AlphaFold* model predicted for the DUF1842 β-barrel domain was sufficiently close to the target structure to serve as a successful molecular-replacement input.

### 3.4. Refinement of atomic structures

Because the form 1 crystals gave the highest resolution diffraction data (from X-rays), a model for the β-sheet-rich domain was refined against these data and then subsequently used as the starting point for model refinements of the other crystal forms (X-ray and electron). This strategy helped to prevent the separation of *R*_free_ and *R*_work_, especially in the case of the microED data, which suffered from low completeness and poor *I*/σ(*I*) and *R*_merge_ statistics.

Importantly, the *Phaser* statistics for molecular-replacement solutions using the refined form 1 crystal structure were much improved over those from *AlphaFold*-predicted models; the form 2 data set gave a *Phaser* LLG value of 624, while the form 3 data set gave a *Phaser* LLG value of 662 (compared with LLGs of 394 and 305 for the *AlphaFold* models). We therefore adopted those solutions as starting points for atomic refinement.

During refinement, we paid close attention to the C-terminal region of the resulting density maps to observe whether the density expected for the C-terminal domain would become visible. In all three forms, large solvent channels were noted adjacent to the C-terminus of the β-barrel domain (Supplementary Fig. S6), which would have allowed possible placement of the small C-terminal segment. Unfortunately, in all crystal forms we observed no meaningful positive density in *F*_o_ − *F*_c_ difference maps in the regions that would have to be occupied by the C-terminal domain. We hypothesize this could be due to proteolysis, as we observed degradation products on SDS–PAGE gels and subsequently in mass spectra from dissolved crystalline samples (Supplementary Fig. S7 and unpublished work). Final refinement for the form 1 model gave an *R* factor of 25.1% and an *R*_free_ of 28.7%. The form 2 model had an *R* factor of 28.4% and an *R*_free_ of 30.7%. The form 3 model had an *R* factor of 27.3% and an *R*_free_ of 33.3%. The structure of the N-terminal β-sheet-rich domain was strongly conserved across all crystal forms and asymmetric units; no protein chain from any of the three crystal forms had a backbone r.m.s.d. above 0.6 Å compared with any other chain (Fig. 5 ▸). There was also close agreement between the refined structures and the *AlphaFold* prediction. The backbone r.m.s.d. values between the experimental structures and the *AlphaFold* model were 0.35 Å for form 1, 0.49 Å for form 2 and 0.46 Å for form 3.

Analyzing the noncrystallographic symmetry of the three crystal forms revealed molecular-packing interfaces that were substantially different (Fig. 5 ▸). As a result, no biologically relevant interfaces could be inferred with confidence. One potentially relevant exception is that the form 2 noncrystallographic interface is present as a crystallographic interface in the form 3 crystals.

### 3.5. Structural analysis

The overall structure of the C-terminal domain of the Q63NT7 protein forms an eight-stranded antiparallel β-barrel. Residues 67–77, corresponding to the amino-acid sequence GPPPRRDGSG, did not appear in any of our electron-density or electrostatic potential maps, and thus were omitted from the structures deposited in the PDB. Polar residues are found covering the exterior surface of the β-barrel, while the interior of the β-barrel is lined with mostly hydrophobic residues, without space for a channel through the barrel. Residues 88–97 form an unusual hydrophobic extended loop, with a conserved structure across crystal forms, which interacts with strand 4 of the β-barrel.

## 4. Discussion and conclusion

In several cases, microED has proven to be an important tool for structural biologists, enabling the extraction of high-resolution structural information from tiny crystals that are unusable for X-ray diffraction. The earliest demonstration of the method on protein crystals was the seminal work on crystals of lysozyme (Shi *et al.*, 2013 ▸). Important early work from Rodriguez and coworkers advanced on these studies and demonstrated the utility of microED in solving the structures of small peptides (Rodriguez *et al.*, 2015 ▸). Other work has demonstrated the utility of the method in solving structures of proteins in cases where structures are already known for proteins that are closely or even distantly related, including ligand-bound or drug-bound forms of proteins (Martynowycz & Gonen, 2021 ▸; Martynowycz *et al.*, 2021 ▸, 2022 ▸; Xu *et al.*, 2019 ▸; Danelius *et al.*, 2023 ▸). Nevertheless, experimental methods for phasing microED data have been elusive (outside of specialized demonstrations from model systems with known structures; Martynowycz *et al.*, 2022 ▸), limiting broader applications of the method. The work presented in this paper adds to the relatively small number of electron diffraction structures of novel proteins. Two recent studies have demonstrated success in phasing microED data using structures of distantly related homologs as search models (Xu *et al.*, 2019 ▸; Clabbers *et al.*, 2021 ▸), and the utility of using *AlphaFold* to generate models of proteins with close homologs of known structure is well documented (Danelius *et al.*, 2023 ▸; Shiriaeva *et al.*, 2023 ▸). As far as we are aware, the current study represents the first folded (globular) protein structure solved by microED whose structure could not be approximated in advance by virtue of a recognizably homologous known structure. We also note that the collection of microED data was challenged by the strong tendency of crystals to adopt a preferred orientation on the EM grid, leading to an incomplete data set, and to less than ideal statistics. This led to some initial uncertainties in assigning a space group and subsequent structure determination. It remains unknown whether diffraction at high tilt angles was inhibited primarily by the increased sample thickness that this entails or by limited ordering of the crystal lattice.

We also present the structure of a new small protein fold and the first from protein family DUF1842. Notably, efforts to obtain structural information on the C-terminal domain from our maps were unsuccessful. Between the two domains, we note the presence of an ∼25-amino-acid linker predicted to form a loop with low sequence conservation across homologues (Fig. 1 ▸ *b*). This could contribute to flexibility of the entire C-terminal region of the protein in the context of the crystal. We also observed several instances of proteolysis in our crystal trays, even with the addition of protease inhibitors and both with and without the sterilizing agent sodium azide added to the crystal drops. The degradation products appear to be composed of prominent fragments of 4–5 and 17–19 kDa based on SDS–PAGE (Supplementary Fig. S7). This could place the cut site directly N-terminal to the C-terminal domain, which did not appear in our crystal structures. The tendency of the protein to undergo proteolysis also lends support to the hypothesis that some part of the C-terminal region of the protein was missing from all three crystal forms, explaining the absence of detectable density in all cases. Considering these data, there could still be unaccounted-for scattering from up to ∼60 amino acids based on the difference between the estimated molecular weight of the abundant bands visible using SDS–PAGE (Supplementary Fig. S7) and the molecular weight of our structures (∼12 kDa). We take this as a possible explanation for the model deficiencies (especially in form 1) and the higher-than-typical refinement *R* values that we ultimately obtained in all three crystal forms.

Despite our initial predictions, based on genomic patterns, that Q63NT7 might be involved in oligomerization via its C-terminal domain, we were unable to observe any evidence of higher order oligomer formation either in solution or in the crystalline form. Our biochemical studies did not support the self-assembly of this protein of unknown function into larger architectures under the conditions tested. Nonetheless, the appearance of a flexible linker to a terminal domain that was unresolved by crystallography is reminiscent of studies on bacterial microcompartment shell proteins (Thompson & Yeates, 2014 ▸), whose genomic patterns were the impetus for the original genomic investigation that identified the IPR014994 domain as a target in the current study (Beeby *et al.*, 2009 ▸). Whether the architecture of the full protein molecule, *i.e.* with the small C-terminal domain intact, might be different remains unclear.

## Supplementary Material

PDB reference: bacterial protein of unknown function, 8t0b

PDB reference: 8t1m

PDB reference: 8t1n

Supplementary Figures and Tables. DOI: 10.1107/S205979832400072X/jb5060sup1.pdf

## Figures and Tables

**Table 1:** Data collection and processing

Crystal form	Form 1	Form 2	Form 3
PDB code	8t0b	8t1n	8t1m
Diffraction source	24-ID-C, APS	Tecnai TF30	24-ID-E, APS
Wavelength	1.4586	0.01969	0.97918
Temperature (K)	100	100	100
Detector	PILATUS 6M-F	TemCam-F416 (4k × 4k)	EIGER X 16M
Crystal-to-detector distance (mm)	200	5280	400
Total rotation range (°)	180	70	70
Rotation per image (°)	0.5	0.85	0.5
Exposure time per image (s)	0.25	10	0.5
No. of crystals	1	4	1
Space group	*P*2_1_	*P*2_1_2_1_2_1_	*P*2_1_2_1_2_1_
*a*, *b*, *c* (Å)	39.5, 40.4, 78.5	40.6, 95.0, 101.5	40.11, 70.82, 94.5
α, β, γ (°)	90, 97.01, 90	90, 90, 90	90, 90, 90
Mosaicity (°)	0.184	0.356 (2, 3, 4), 0.157 (7)	0.176 (0–70), 0.188 (140–210)
Resolution range† (Å)	77.9–2.1 (2.15–2.10)	35.0–3.0 (3.10–3.02)	47.3–3.00 (3.08–3.00)
Total No. of reflections	45764	39900	13935
No. of unique reflections	25176	4846	5084
Completeness† (%)	89.1 (82.9)	58.8 (44.2)	87.9 (88.8)
Multiplicity	1.8	11.7	2.74
〈*I*/σ(*I*)〉†	8.9 (1.4)	5.6 (2.5)	4.4 (2.3)
CC_1/2_ †	99.9 (76.6)	91.2 (14)	97.2 (29.9)
*R*_r.i.m._ †	0.059 (0.701)	0.386 (0.501)	0.272 (1.36)
Overall *B* factor from Wilson plot (Å^2^)	45.7	24.0	56.3

† Values in parentheses are for the outer shell.

**Table 2:** Refinement statistics Values in parentheses are for the outer shell.

Crystal form	Form 1	Form 2	Form 3
Resolution range (Å)	77.9–2.10 (2.17–2.10)	35.0–3.02 (3.80–3.02)	47.25–3.00 (3.30–3.00)
Completeness (%)	95.0 (90)	58.8 (57)	87.7 (89)
No. of reflections, working set	12543	4581	4562
No. of reflections, test set	1395	242	508
*R*_work_ (%)	25.1 (34.3)	28.3 (32.3)	27.4 (32.9)
*R*_free_ (%)	28.7 (35.0)	30.7 (34.5)	33.3 (42.2)
No. of non-H atoms			
Protein	1752	1740	1709
Ions	0	0	0
Ligands	0	0	0
Waters	11	0	0
Total	1763	1740	1709
R.m.s.d., bond lengths (Å)	0.008	0.014	0.011
R.m.s.d., bond angles (°)	0.95	1.59	1.44
Average *B* factors (Å^2^)			
Protein	50.0	12.35	49.57
Waters	48.7	N/A	N/A
Ramachandran outliers (%)	0	0	0
Ramachandra favored (%)	96.1	95.7	96.9
Unmodelled/incomplete residues (%)	7.6	7.6	8.5
