# Supplementary material for: AlphaFold-assisted structure determination of a bacterial protein of unknown function using X-ray and electron crystallography
Source: Acta Crystallogr D Struct Biol. 2024 Mar 7;80(Pt 4):270–8. doi: 10.1107/S205979832400072X (PMC10994174; doi:10.1107/S205979832400072X)
Supplement: Supplementary file 1 [file d-80-00270-sup1.pdf]

STRUCTURAL BIOLOGY

**Volume 80 (2024)**

**Supporting information for article:**

# *AlphaFold*-assisted structure determination of a bacterial protein of unknown function using X-ray and electron crystallography

**Justin E. Miller, Matthew P. Agdanowski, Joshua L. Dolinsky, Michael R. Sawaya, Duilio Cascio, Jose A. Rodriguez and Todd Yeates**

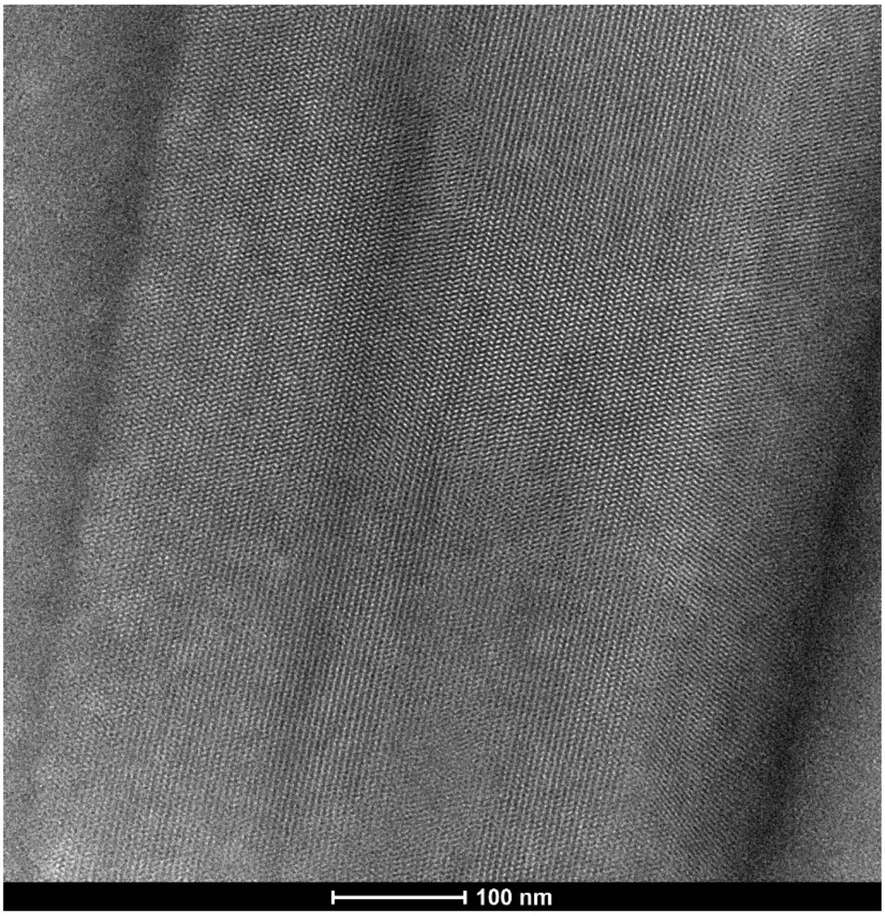


**Figure S1** Negatively stained Q63NT7 crystal visualized on a Talos F200C electron microscope.

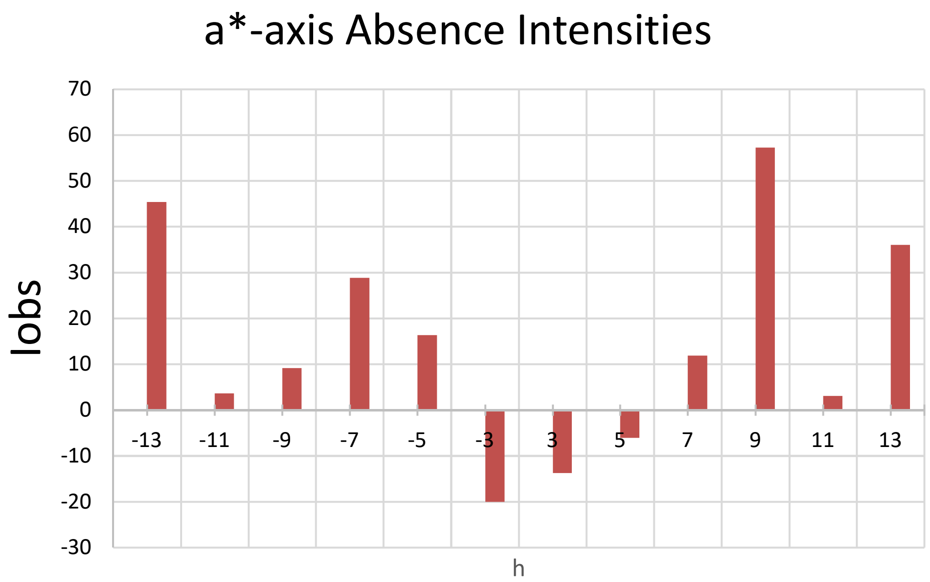


| | |
|---|---|
| n | 12 |
| x̄ | 14.35 |
| SD | 23.66 |
| SEM | 6.83 |

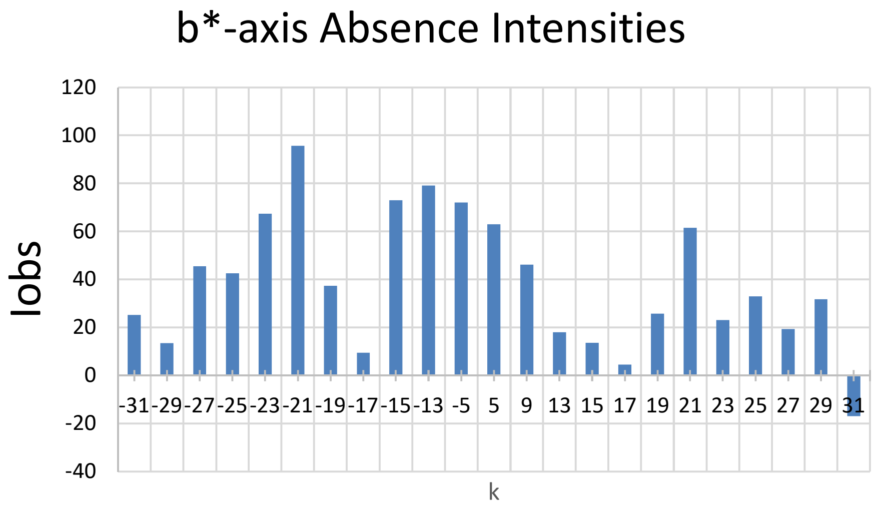


| | |
|---|---|
| n | 23 |
| x̄ | 38.46 |
| SD | 27.87 |
| SEM | 5.81 |

**Figure S2** Histogram representations of intensities of systematic absences in microED data collected from form 2 crystals. The SEM value is the uncertainty in the mean value (equal to SD/sqrt(n)). Along b*, the average intensities of odd index reflections substantially exceed the uncertainty in the mean, which suggests the presence of dynamical scattering. For comparison, the mean intensities for (2n, 0, 0) and (0, 2n, 0) reflections were 94.7 and 107.3, respectively over the same index ranges as above.

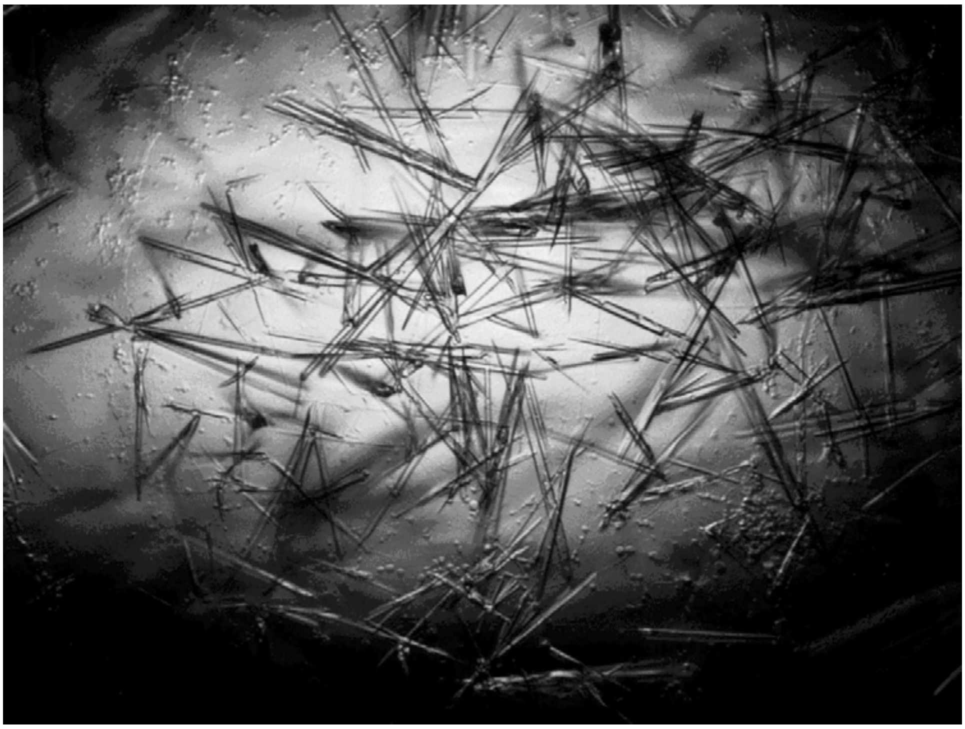

**Figure S3** Light microscope image of form 3 crystals.

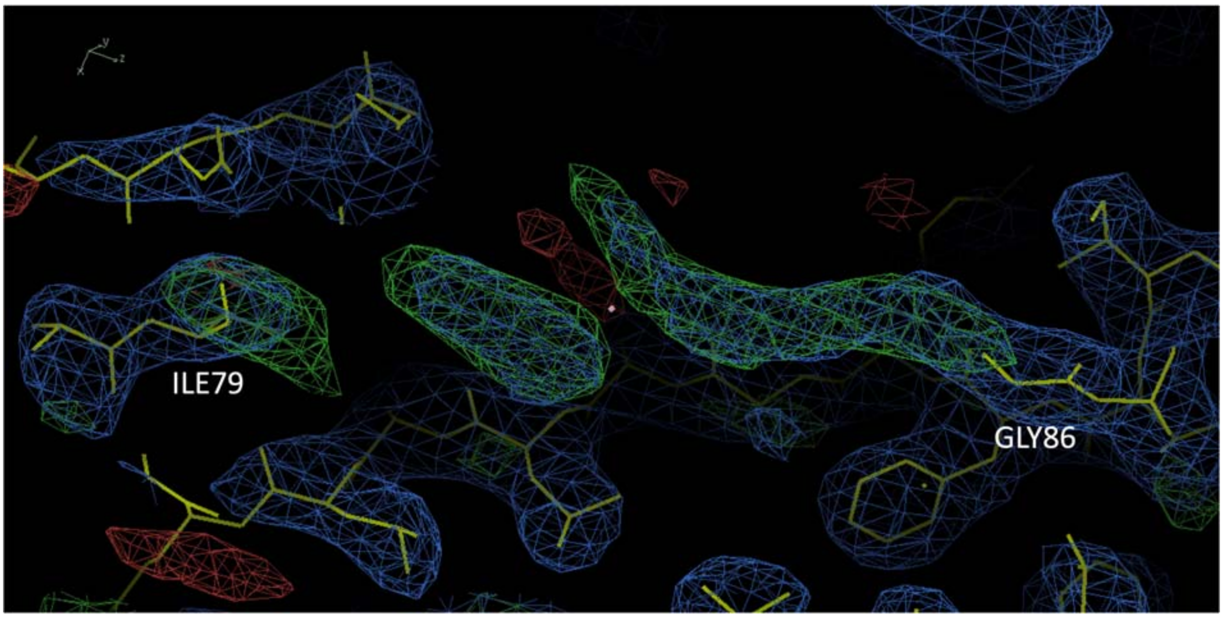


**Figure S4** A Micro-ED omit-map confirms the correct molecular replacement solution when using the AlphaFold search model on form 2 diffraction data. Six residues from beta strand 5 were excluded from the search model, and density appears for this missing segment in an $F_o$-$F_c$ map calculated using model

phases. Molecular replacement search model shown as atomic model in yellow, green density corresponds to positive density in $F_o$-$F_c$ map.

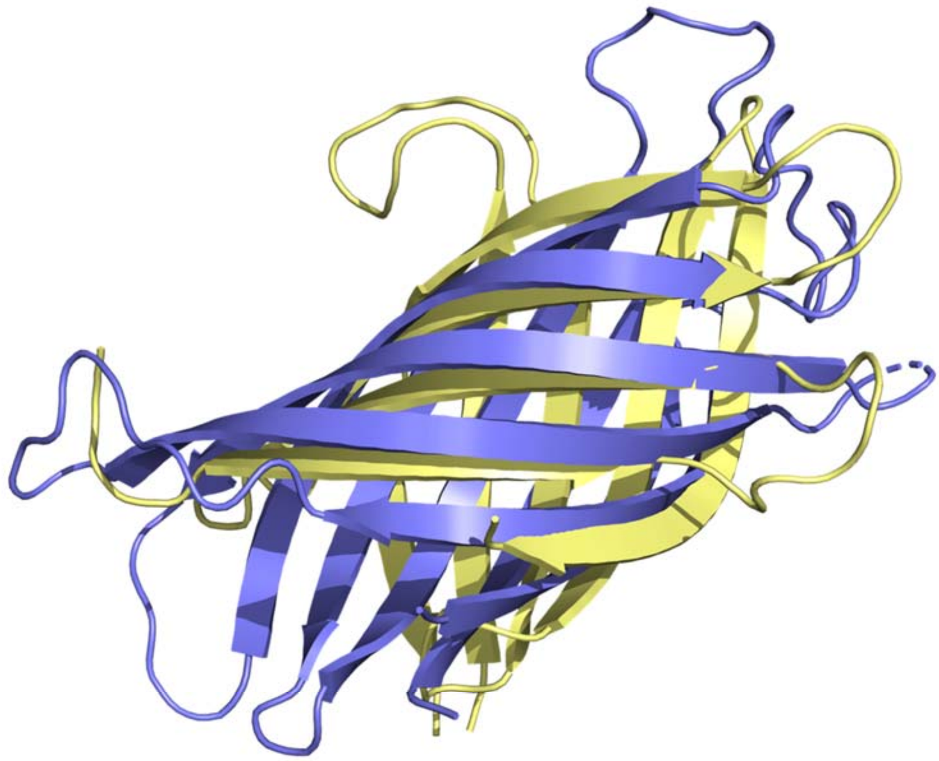

**Figure S5** Comparison of the closest identifiable homolog of known structure (PDB 2erv) with the experimental structure of protein Q63NT7. The 2erv structure is shown as a cartoon in purple overlayed with the form 1 crystal structure of Q63NT7 (yellow).

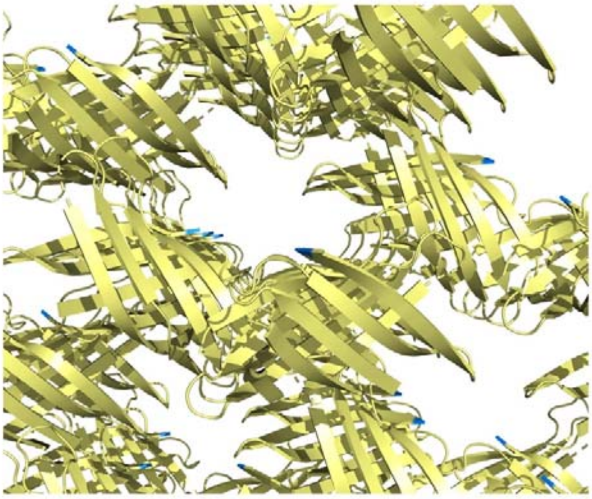

**Figure S6** Crystal packing for the form 1 crystal of the Q63NT7 protein reveals solvent channels at the C-terminus of the β-barrel domain.

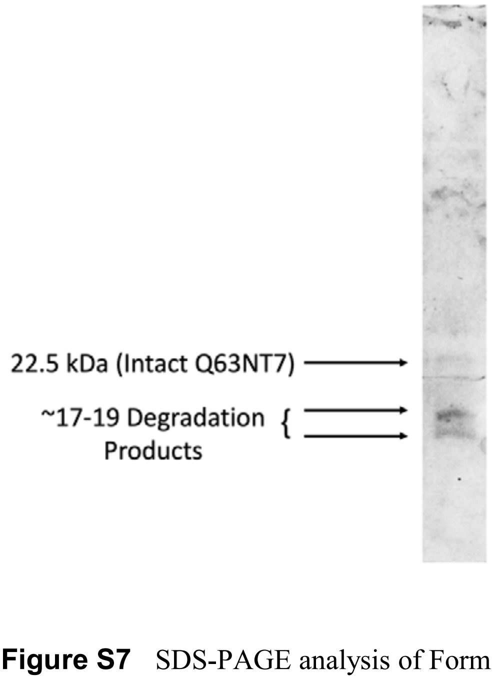


**Figure S7** SDS-PAGE analysis of Form 3 crystals reveals prominent degradation products for the Q63NT7 protein.

**Table S1** Macromolecule Production

| | |
|---|---|
| Source Organism | *Burkholderia pseudomallei* |
| DNA Source | Synthetic |
| Expression vector | Pet 22b (+) |
| Plasmid Construction method | Gibson assembly |
| Expression host | *Escherichia coli* (BL21 (DE3)) |
| Expression details | Autoinduction(Studier, 2005) |
| Complete amino-acid sequence of the protein produced:<br>MSEDLRVGLFPVRYLVGTGLPGAPQLVLDLMVDTVDHSVVGRAAVSQAVSPPLNFHADVWG<br>SYVFRLGPPPRRDGSGAIVQISLQGNQGGPQSNSMITFYGELLLKGDGKTGVASYRYYSNGSW<br>HEVENVPVKADPELVPIEPGPVIGQSSMSAIGSAAMYGVAIQSAAASGDLAHMRTLSAYARQQ<br>LESRDEIAAALSELKAEIAKLESRQHHHHHH | |

**Table S2** Crystallization Form 1 Crystals

| | |
|---|---|
| Method | Hanging drop |
| Plate type | 96 well |
| Temperature (°C) | 20 |
| Protein Concentration | 20 mg/ml |
| Buffer composition of protein | 100mM BisTris pH 5.5, 25% PEG 3350 |
| Volume and ratio of drop | 2:1 |
| Drop setting | SPT LabTech Mosquito |
| Seeding | No |

**Table S3** Crystallization Form 2 Crystals

| | |
|---|---|
| Method | Hanging drop |
| Plate type | 96 well |
| Temperature (°C) | 20 |
| Protein Concentration | 20 mg/ml |
| Buffer composition of protein | 100mM BisTris pH 5.5, 100mM Ammonium Acetate 17% PEG 10,000 |
| Volume and ratio of drop | 2:1 |
| Drop setting | SPT LabTech Mosquito |
| Seeding | No |

**Table S4** Crystallization Form 3 Crystals

| | |
|---|---|
| Method | Hanging drop |
| Plate type | 96 well |
| Temperature (°C) | 20 |
| Protein Concentration | 100 mg/ml |
| Buffer composition of protein | 100mM TRIS HCl pH 8.5, 150mM MgCl, 12.5% PEG 8000 |
| Volume and ratio of drop | 1:1 |
| Drop setting | SPT LabTech Mosquito |
| Seeding | No |
